# Impact of Manganese, Copper and Zinc Ions on the Transcriptome of the Nosocomial Pathogen *Enterococcus faecalis* V583

**DOI:** 10.1371/journal.pone.0026519

**Published:** 2011-10-28

**Authors:** Marta Coelho Abrantes, Maria de Fátima Lopes, Jan Kok

**Affiliations:** 1 Laboratory of Stress by Antibiotics and Virulence of Enterococci, Instituto de Tecnologia Química e Biológica, Universidade Nova de Lisboa, Oeiras, Portugal; 2 Department of Molecular Genetics, University of Groningen, Groningen, The Netherlands; 3 Instituto de Biologia Experimental e Tecnológica, Oeiras, Portugal; Newcastle University, United Kingdom

## Abstract

Mechanisms that enable *Enterococcus* to cope with different environmental stresses and their contribution to the switch from commensalism to pathogenicity of this organism are still poorly understood. Maintenance of intracellular homeostasis of metal ions is crucial for survival of these bacteria. In particular Zn^2+^, Mn^2+^ and Cu^2+^ are very important metal ions as they are co-factors of many enzymes, are involved in oxidative stress defense and have a role in the immune system of the host. Their concentrations inside the human body vary hugely, which makes it imperative for *Enterococcus* to fine-tune metal ion homeostasis in order to survive inside the host and colonize it. Little is known about metal regulation in *Enterococcus faecalis*. Here we present the first genome-wide description of gene expression of *E. faecalis* V583 growing in the presence of high concentrations of zinc, manganese or copper ions. The DNA microarray experiments revealed that mostly transporters are involved in the responses of *E. faecalis* to prolonged exposure to high metal concentrations although genes involved in cellular processes, in energy and amino acid metabolisms and genes related to the cell envelope also seem to play important roles.

## Introduction

Maintenance of intracellular homeostasis of metal ions is crucial for survival of bacteria, particularly for appropriate transcriptional control of regulatory networks that govern gene expression and for virulence. Thus, mechanisms for metal ion homeostasis or, more specifically, metal ion transport may constitute major adaptations to intracellular survival and replication among pathogenic bacteria [1]. Of distinct relevance are zinc, copper and manganese ions, not only as components of many proteins and co-factors in enzymatic reactions, but also for their toxicity to bacterial cells when present above certain concentrations. Within the host, pathogens can come across variable concentrations of these metals which demands a precise transcriptional control of genes coding for transporters (responsible for metal uptake and efflux) or proteins involved in metal ion storage. In fact, the total zinc concentration in serum and in gastric juice is similar (13.8 µM and 13 µM, respectively [2]; [3]), but in saliva and in the lungs the total concentration of this metal can reach 133.3 µM and 229 µM, respectively [2], [4]. Zn^2+^ has a strong influence on the immune function of the human body [5], [6], [7]. In general, low levels of zinc lead to decreased performance of the immune system, while physiologically normal concentrations secure its normal functioning [6], [8]. A high concentration of Zn^2+^ (0.1 mM) may even activate certain immune cells [8]. Moreover, zinc levels in the human body are increased during inflammation [5], [7]. Manganese is another important trace metal required in numerous cellular processes, including metabolism and oxidative stress defense [9]. Manganese may protect against oxygen reactive species and increase the fitness of cells by minimizing energy expenditure on the synthesis of a defense regulon [10]. The total concentration of this metal is 1000-fold higher in secretions such as saliva (36.2 µM, [4]) than it is inside the human body, for example in blood (11.6 nM, [2]) or in urine, where Mn^2+^ levels are also in the nanomolar range [11]. Thus, manganese ions become a potential signal by which bacteria can sense a shift from a mucosal environment to a more invasive site. Copper is an essential trace element required by most organisms as a cofactor for many catabolic pathways and electron transport. However, copper is toxic to cells at concentrations higher than physiological levels (16 µM in serum, [2]) and excess copper avidly binds to many biomolecules such as proteins, lipids and nucleic acids, regardless of its valence state [12]. Thus, exposure to metals with redox properties such as copper [13] is a double-edged sword, for these properties render them highly toxic through interference with the functioning of intracellular macromolecules and because they can generate toxic free radicals through the Fenton reaction [14].

*Enterococcus faecalis* is a Gram-positive bacterium with a dual nature, as it is present in the human digestive tract as a commensal organism, but is also frequently the cause of nosocomial infections. Mechanisms and factors involved in the switch from commensalism to pathogenicity of these bacteria remain unclear despite the fact that some virulence-associated genes have been identified [15], [16], [17], [18]. Information about environmental stresses and their contribution to the switch to pathogenicity is still scarce. Since bacterial responses to stress often coincide with increased virulence [19], [20], a number of studies have been conducted recently where *E. faecalis* V583 gene expression was examined in different conditions mimicking the various environments in the host. Conditions such as the ones found in the gastro-intestinal tract were represented with sodium dodecyl sulfate (SDS) and bovine bile (BB) stresses in V583 strain [21]; other environments such as blood and urine were also studied [22], [23]. Another study probed the expression of virulence related genes in *E. faecalis* OG1RF submitted to several sub-lethal stresses [24].

Nevertheless, little is known on how *E. faecalis* is able to cope with changes in metal concentrations in the host and assure its own metal regulation. In other Gram-positive pathogens, several transcriptional studies with metal ions have been conducted to gain more knowledge on metal homeostasis in those organisms [25], [26], [27], [28], [29]. Different studies in *Enterococcus hirae* led to the description and characterization of copper regulation by the *cop* operon [30], [31], [32]. More recently, a transcriptomic study on copper stress was performed in *E. faecalis* OG1RF strain, which helped to identify other regulators putatively involved in copper homeostasis through the *cop* operon [33]. Regarding manganese homeostasis in *E. faecalis* JH2-2, it was suggested that the *efaCBA* operon, encoding the virulence factor EfaA, is regulated by EfaR in a manganese-dependent way [34]. Nothing has yet been described on zinc regulation in this species. The poor knowledge on how these bacteria cope with different metals in the environment, being able to survive and cause infection, propels our work on the transcriptional response to metal stresses of *E. faecalis* V583, a vancomycin resistant clinical isolate and the first *E. faecalis* strain to have its genome sequenced. We performed DNA microarray experiments on *E. faecalis* V583 grown in the presence of Zn^2+^, Mn^2+^ and Cu^2+^ and give the first description of *E. faecalis* V583 transcriptomes under high concentrations of these metal ions. Such studies will help unravel some important mechanisms that are involved in metal regulation in this organism.

## Materials and Methods

### Media and growth conditions

*E. faecalis* strain V583 [13] was grown in M17 with 0.5% glucose (GM17) and metal solution when required. GM17 contains 2.0 µM Mn^2+^, 8.1 µM Zn^2+^ [25] and 2.1 mM SO_4_^2−^ [35]. Data for Cu^2+^ are not available. Metal sensitivity tests were performed as follows: Growth of *E. faecalis* V583 in the presence of various concentrations of each metal was followed in Schott GL18 glass tubes (Schott, Elmsford, NY, USA) and OD_600_ was read using a Spectronic 21D (Milton Roy, Pont-Saint-Pierre, France). Sterile metal stock solutions were prepared in water and stored at −20°C.

### Transcriptome experiments

DNA microarray experiments were performed essentially as described previously [36], [37]. RNA was isolated from 30 ml of *E. faecalis* V583 culture grown until mid-exponential phase in GM17 with either (added-metal concentrations): ZnSO_4_ (0, 4 mM), MnSO_4_ (0, 0.4 mM) or CuSO_4_ (0, 0.05 mM). Cells were harvested by centrifugation for 1 min at 10000 rpm at room temperature. Cell pellets were immediately frozen in liquid nitrogen and stored at −80°C. Initial steps of RNA isolation were performed as previously described [37]. Isolation was completed with the Roche High Pure RNA isolation kit (Roche Diagnostics GmbH, Mannheim, Germany). cDNA synthesis and indirect labeling with Cy-3-dCTP and Cy-5-dCTP were performed as reported [37]. Labeled cDNA was mixed and hybridized on glass slides carrying 70-mer oligonucleotides for 3160 genes of *E. faecalis* V583 [38]. Hybridization (16 h at 45°C) was performed with Ambion Slidehyb #1 hybridization buffer (Ambion Europe Ltd., Huntingdon, United Kingdom). Slides were scanned in a GeneTac LS IV confocal laser scanner (Genomics Solutions, Huntingdon, United Kingdom).

### DNA microarray data analysis

Slide images were analyzed using GenePix Pro 6.0 software. Processing and normalization (LOWESS spotpin-based) of slides was done with the in-house developed *MicroPrep* software [36]. DNA microarray data were obtained from at least two independent biological replicates and a dye swap hybridized to three glass slides. Expression ratios were calculated from the measurements of nine spots per gene. Differential expression of genes was determined as previously described [37]. A gene was considered differentially expressed when the *p* value was ≤0.0001 and the ratio≥|1.5|. Data obtained were further analyzed and grouped in functional categories according to the JCVI (J. Craig Venter Institute) website (http://cmr.jcvi.org). A Venn diagram was used to visualize and compare the results obtained for the three metal experiments.

Data were uploaded in GEO with accession numbers # GSE30947, GSE30948 and GSE30949.

### Semi-quantitative reverse transcriptase (sqRT)-PCR

Expression of selected genes in *E. faecalis* V583 was studied by sqRT-PCR, to confirm results obtained in the DNA microarray experiments. The strain was grown in GM17, with and without one of the following metal solutions: ZnCl_2_ (at 4 mM added-metal concentration), MnCl_2_ (at 0.4 mM added-metal concentration) and CuSO_4_ (at 0.05 mM added-metal concentration). Cells were grown until mid-exponential phase; RNA was then extracted with Qiagen RNeasy Mini Kit (Qiagen GmbH, Hilden, Germany), following the manufacturers' procedure. cDNA synthesis was done with the Roche Transcriptor High Fidelity cDNA synthesis Kit (Roche Diagnostics GmbH). For these reactions, cDNA dilutions 10^0^, 10^−1^ and 10^−2^ were used. PCR products were obtained with Finnzymes® Taq DNA polymerase MasterMix (Finnzymes OY, Espoo, Finland). Primers used are described in Table 1.

**Table 1:** List of primers used in this study.

Gene	Sequence (5′ to 3′)	Amplicon size (bp)
*ef0575*	TTTTGTGTACCATACAATCG	813
	AAGTGAAAGTCATACAGACC	
*ef0758*	AGGCTTTGGAGATACGTATCG	1549
	TTGAGACACGGTGAGCATAGC	
*mntH2*	TATTGCAAAACGAAAGAAGG	1620
	TAACCTCCTCTACTTGTTGC	
*ef1400*	GACACTGGAAGTTGAATCAGG	1911
	ACCAACATCAGCAAACACTGC	
*efaC*	CCTTATACTGATTTTAAGGC	814
	AAACCATCAATAAATGCAGC	
*ef0575* promoter region	GAGAAGAATTCAATGGTCTTCCCATGTATTTAGG	548
	AAGCAGGATCCATTTTCCAGCACCATTTGGACC	

### Transcriptional *lacZ* fusion construction

In order to observe the effect of addition of metals in gene expression through β-galactosidase assays, a transcriptional fusion with the *E. coli lacZ* gene was constructed in plasmid pILORI4 [39]. Primer pair P*ef0575*-1/P*ef0575*-2 was used to generate a PCR fragment spanning the whole upstream region of *ef0575* (Table 1). The fragment was cloned in EcoRI/BamHI site of pILORI4, which was subsequently introduced in the strain VE14089, corresponding to strain V583 cured of its plasmids.

### β-galactosidase assays

The procedure was based on the assay described by Miller [40]. Cells were grown in GM17 with erythromycin 50 µg/ml and metals. Metal solutions were used in the following added-metal concentrations ZnCl_2_: 0 and 4 mM, MnCl_2_: 0 and 0.4 mM, CuSO_4_: 0 and 0.05 mM, Mid-log cells were spun down and frozen in liquid nitrogen to be used later. Frozen cell pellets were resuspended in the same volume of Z buffer (Na_2_HPO_4_ 0.06 M, NaH_2_PO_4_ 0.04 M, KCl 0.01 M, MgSO_4_ 0.001 M and β-mercaptoethanol 0.05 M, pH 7.0), and the OD_600_ was measured. Diluted cells were permeabilized with 100 µl of chloroform and 50 µl of 0.1% SDS. Reactions were started with the addition of 200 µl of 2-Nitrophenyl β-D-galactopyranoside (Sigma-Aldrich Chemie GmbH, Steinheim, Germany) (4 mg/ml in 0.1 M phosphate buffer, pH 7.0) and stopped with 500 µl of 1 M Na_2_CO_3_. Cells were centrifuged for 5 min at 14000 rpm and the OD at 420 and 550 nm were recorded. Activity of LacZ (in Miller units) was calculated according to Miller [40]. These assays were performed as three independent replicates.

## Results

**Figure 1:**
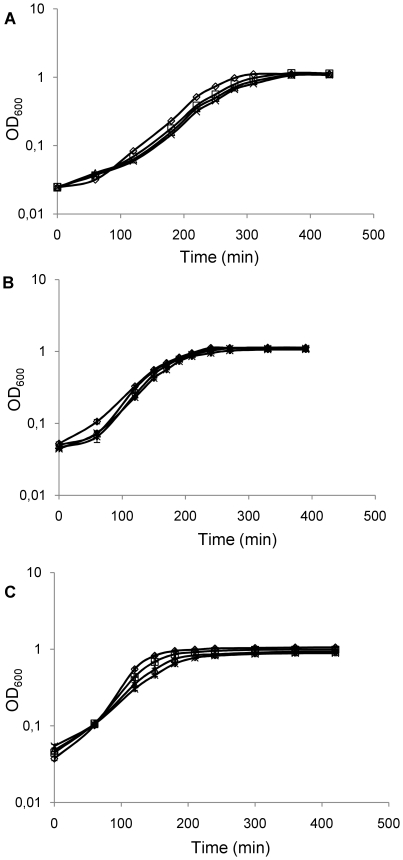
*E. faecalis* V583 growth in GM17 with and without added metals. **A**. *E. faecalis* V583 growth in GM17 (empty diamonds) and GM17 with 4 mM ZnSO_4_ (empty squares), 5 mM ZnSO_4_ (empty triangles) or 6 mM ZnSO_4_ (crosses); **B**. *E. faecalis* V583 growth in GM17 (empty diamonds) and GM17 with 0.4 mM MnSO_4_ (empty triangles), 1 mM MnSO_4_ (crosses) or 2 mM MnSO_4_ (asterisks); **C**. *E. faecalis* V583 growth in GM17 (empty diamonds) and GM17 with 0.05 mM CuSO_4_ (empty squares), 0.075 mM CuSO_4_ (empty triangles) and 0.100 mM CuSO_4_ (crosses). Indicated are the added metal concentrations. All experiments were performed in triplicate.

We have studied the transcriptional response of *E. faecalis* V583 to different metal stresses using a genome-wide DNA microarray approach. By allowing the cells to grow until mid-exponential phase in the presence of high concentrations of metals, we were able to investigate the effects of prolonged exposure to these stresses. To simplify, we will use the term “metal stress” when referring to the prolonged exposure to high concentration of metals. In each metal experiment, the transcriptomes of V583 grown in GM17 with added metal and in GM17 without added metal were compared. To find the appropriate concentrations of metal solutions to use in these experiments, growth of *E. faecalis* V583 in GM17 with different metal concentrations was recorded. For DNA microarray experiments added-metal concentrations of 4 mM Zn^2+^, 0.4 mM Mn^2+^ and 0.05 mM Cu^2+^ were chosen as these only slightly affected cell growth, causing no more than a 30 min delay in growth, as shown in Figure 1.

**Figure 2:**
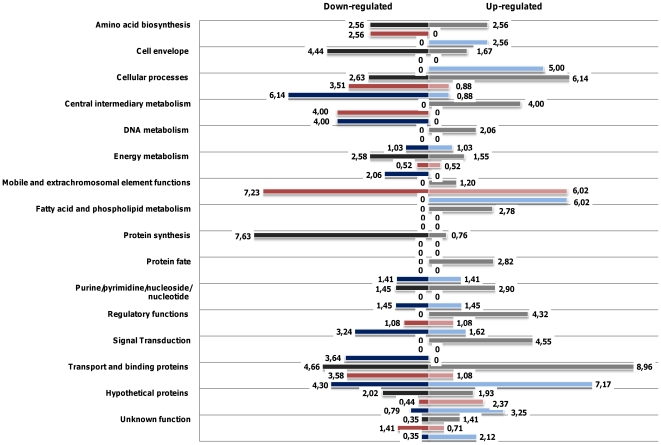
Representation of *E. faecalis* V583 transcriptome responses to metal stress by functional categories. Representation of the weight of each functional category in the transcriptome responses of *E. faecalis* V583 to high concentrations of Zn^2+^, Mn^2+^ or Cu^2+^. The weight represents the percentage of genes differentially expressed in each category relative to the total number of predicted genes in that category in the genome of V583. Zinc experiments – black/grey bars; manganese experiments - dark pink/light pink; copper experiments – dark blue/light blue. The weight in percentage is given at the sides of the bars.

The DNA microarray results using the three metal concentrations are represented in Figure 2. In order to see the relevance of each gene functional category in response to the imposed metal stresses, we have represented the weight of each category by dividing the number of genes differentially expressed in each category by the total number of predicted genes in that category in the genome of *E. faecalis* V583. Most of the genes that were differentially expressed in the presence of any of the three metals code for hypothetical proteins. However, this group of genes does not assume a particular relevance in response to the imposed metal stresses, as they represent only around 5% of all hypothetical proteins in the V583 genome. Other functional categories, particularly those including genes encoding transport and binding proteins, reaching weight values of 15%, are more important for growth in the presence of these high metal concentrations.

Genes encoded by the plasmids pTEF1, pTEF2 or pTEF3 present in *E. faecalis* V583 [13] were not differentially expressed in any of these DNA microarray experiments.

### Zn^2+^-regulated genes

Genes differentially expressed in the presence of 4 mM ZnSO_4_ represent ca. 4.8% of all V583 genes (Table S1). Most of them are up-regulated (87 genes). Genes putatively involved in transport represent approximately 25% of all differentially expressed genes; most of these are induced in the presence of Zn^2+^. Of these transport related genes 45% encode proteins described as metal transporters, according to the National Center for Biotechnology Information (NCBI, http://www.ncbi.nlm.nih.gov). About 16% of all differentially expressed genes are present in mobile genetic regions, including ten up-regulated genes in the pathogenicity island (PAI). Several metabolic pathways (amino acid, purine, glycerophospholipid, energy and vitamin metabolism and also peptidoglycan biosynthesis) were affected by zinc stress, mostly by up-regulation of some of the genes involved in those pathways. Seven genes related to the cell envelope were repressed in the presence of zinc stress; three of these genes are involved either in teichoic acid biosynthesis (*ef2486* and *ef2487*) or in peptidoglycan biosynthesis (*ef0993*). In addition, genetic information processing was altered, mainly by repression of nine ribosomal genes, which may be an indication of a slight difference in growth in the presence of zinc. Ten genes involved in cellular processes were also differentially expressed. Amongst these are genes encoding stress related proteins (Gls24, EF0781, EF1058 and EF1084), adhesion lipoproteins (EF0055, EF0577 and EfaA) and a putative aggregation substance (EF0149) proposed to be related to virulence [41]. Most of these genes were up-regulated, suggesting an important role in adjustment to this stress. All putative regulatory genes that showed differential expression under zinc stress were up-regulated. Six genes involved in signal transduction were up-regulated in the presence of zinc. These include genes encoding a PTS system involved in the mannose pathway (EF0552-3), KdpDE (constituting the two-component system Ehk-Err12 reported to be involved in potassium transport [42]), and the two-component system Err-Ehk06 (EF1260-1). Overall, eight genes differentially expressed in the presence of zinc stress were proposed to be involved in virulence.

ZnuABC is a zinc uptake system first described in *E. coli* [43]. Its homologue in V583 (EF0055–EF0057) is encoded by the most down-regulated operon in the experiments with zinc. An operon encoding a cadmium translocating P-type ATPase and a putative SapB family protein (*ef0758*, *ef0759*), together with another cadmium translocating P-type ATPase encoding gene (*ef1400*), were the highest up-regulated genes under zinc stress. The *glnA* and *glnR* genes, involved in amino acid metabolism, were induced. The *fatB-ceuDCB* cluster (*ef3082–ef3085*), proposed to be involved in iron transport and virulence [41], was up-regulated.

### Mn^2+^-regulated genes

During growth in the presence of 0.4 mM manganese, approximately 2.4% of all genes of *E. faecalis* V583 (Table S1) were differentially expressed, most of them being up-regulated (41 genes). Phage encoding genes are apparently very sensitive to manganese stress as they constitute 64% of all differentially expressed genes and 88% of the up-regulated genes under the conditions tested. Approximately 73% of all genes (55 genes) showing differential expression are present in mobile genetic regions. Manganese induced more changes in gene expression of mobile genetic regions than zinc or copper.

Transporters also seem to play important roles in manganese homeostasis. Most of the transport systems are probably related to Mn^2+^ uptake under normal conditions, as the corresponding genes were mainly down-regulation in the presence of the high concentration of manganese applied in this experiment. More than half of the transporter genes differentially expressed in these manganese experiments are described as encoding metal transporters. Other transport-related genes were down-regulated, namely an operon coding for two ABC transporters (*ef0583-4*). One cation efflux family protein showing high homology with the Mn^2+^ efflux system protein MntE in *Streptococcus pneumoniae* [44], [45] is encoded by *ef0859* and it was up-regulated. Four genes that code for transcriptional regulators (*ef0107*, *ef0578*, *ef0579* and *ef2138*) were differentially expressed. Four genes involved in cellular processes were down-regulated, of which two, *ef0577* and *efaA*, encode adhesion lipoproteins and are putatively involved in virulence [41]; the remaining two encode a universal stress protein mentioned above (EF1058) and AhpC, an alkyl hydroperoxide reductase potentially involved in oxidative stress response [41].

### Cu^2+^-regulated genes

Genes differentially expressed in the presence of 0.05 mM CuSO_4_ represent ca. 4.1% of all V583 genes (Table S1); most of them were up-regulated (85 genes). In the presence of copper the lowest percentage of differentially expressed genes in mobile genetic regions was seen, namely approximately 10% down-regulated genes.

Transport related genes apparently also play an important role during growth in the presence of this metal as they represent 24% of all affected genes. Most of these were up-regulated. Nine genes related to the cell wall, including the *dlt* operon and *ef0559* which are involved in cell wall biosynthesis, were induced. Genes involved in cellular processes and signaling were mostly down-regulated. In this group are genes that encode two adhesion lipoproteins (EF0577 and EfaA) and one putative aggregation substance (EF0149) proposed to be related to virulence [41] and also seen responding to the other two metals, and four stress response related proteins (EF0298, EF1058, EF1076 and KatA). The *cop* operon (*ef0297-9*), described as being responsible for copper uptake, availability and export in *E. hirae* [30], was the highest induced operon, as previously observed for *E. faecalis* OG1RF [33]. The genes *kdpA* and *kdpB*, involved in potassium transport [42], were also up-regulated. An operon coding for V-type ATP enzymes (EF1492–EF1500), proposed to be related to osmotic stress [41], was also up-regulated. Another operon that codes for two ABC transporters, a hypothetical protein and a GntR family transcriptional regulator (*ef1673-76*) was highly up-regulated. Three genes proposed to be related to virulence [41], encoding a LemA family protein with unknown function (EF0468), a cell-envelope associated acid phosphatase (EF3245) and a cell wall surface anchor family protein (EF3314) were up-regulated.

In order to confirm the transcriptome results, the expression in GM17 with or without added metals of five genes was studied by sqRT-PCR. In these experiments the expression of these five genes showed the same tendency as observed in the DNA microarray experiments with high concentrations of zinc, manganese and copper ions. Results for genes *ef0758* and *mntH2* are shown in Figure S1. The same effects of addition of metals were also observed on β-galactosidase assays with a P*ef0575*::*lacZ* fusion, represented on Figure S2.

### Common genes

**Figure 3:**
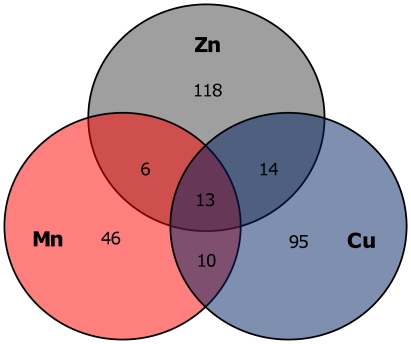
Distribution of differentially expressed genes in the presence of high concentrations of metals. Distribution of *E. faecalis* V583 genes differentially expressed in the presence of 4 mM Zn^2+^ (Zn), 0.4 mM Mn^2+^ (Mn) and 0.05 mM Cu^2+^ (Cu). Venn diagram showing the number of unique and common differentially expressed genes in the three metal DNA microarray experiments.

The Venn diagram in Figure 3 shows that most of the genes that were differentially expressed in the presence of one of the metals were specific for that metal; only a few genes respond to the presence of two metals while a small number of genes respond to all three metals.

Differentially expressed genes common to the three metal DNA microarray experiments are shown in Table 2. Most of these genes code for putative metal transporters and some are involved in energy or amino acid metabolism. One such gene, *arcA* (*ef0104*), from the arginine metabolism, was repressed under zinc or copper stress and induced in the presence of manganese. In the same operon, *ef0107-108* were also down-regulated under copper stress and overexpressed with manganese. An operon encoding two ABC transporters and an aspartate aminotransferase (*ef0893-91*) was repressed under manganese stress and induced in the presence of copper; the first gene was also repressed under zinc stress. With the exception of *arcA* and *ef0893*, all genes common to the three metal stress conditions were overexpressed in the presence of zinc and down-regulated in the presence of manganese and copper. Two homologous systems, *efaCBA* and *ef0575-78*, were the most differentially expressed in this group of common genes. Each of these systems codes for two putative cationic ABC transporters and an adhesion lipoprotein. The last system also encodes a putative iron-dependent repressor family protein (EF0578) while immediately downstream another regulator is encoded, EF0579, a TetR family regulator of which the gene was also differentially expressed under all three metal conditions. The *efaCBA* operon has been described to code for a manganese scavenging system [34] and includes the virulence factor EfaA [15]. Also among the group of common genes are *mntH2*, coding for a putative Mn^2+^/Fe^2+^ transporter, and *ef1058*, encoding a putative universal stress protein.

**Table 2:** Genes differentially expressed in *E. faecalis* V583 grown in the presence of ZnSO_4_, MnSO_4_ or CuSO_4_.

Gene	Fold change^a^			Description^b^
	Zn	Mn	Cu	
***ef0104 (arcA)***	−1.59	1.99	−4.67	arginine deiminase
***ef0575***	2.64	−8.21	−1.85	cationic ABC transporter, ATP-binding protein
***ef0576***	3.97	−7.04	−1.86	cationic ABC transporter, permease protein
***ef0577***	3.85	−5.39	−1.67	adhesion lipoprotein
***ef0578***	6.40	−4.77	−1.49	helix-turn-helix, iron-dependent repressor family
***ef0579***	2.62	−1.90	−1.27	transcriptional regulator, putative
***ef0893***	−1.52	−1.72	2.18	amino acid ABC transporter, amino acid binding/ permease protein
***ef1057 (mntH2)***	2.97	−7.69	−3.10	Mn^2+^/Fe^2+^ transporter, NRAMP family
***ef1058***	5.36	−5.80	−3.78	universal stress protein family
***ef2074 (efaC)***	2.94	−16.58	−2.24	ABC transporter, ATP-binding protein
***ef2075 (efaB)***	4.10	−13.14	−2.01	ABC transporter, permease protein
***ef2076 (efaA)***	4.02	−11.54	−2.03	endocarditis specific antigen

a Metals used (added-metal concentrations in GM17): 4 mM ZnSO_4_, 0.4 mM MnSO_4_ or 0.05 mM CuSO_4_; fold change represents the gene expression in the presence of the indicated metal ion over the gene expression with no added metal. Genes were considered differentially expressed when fold changes were ≤|1.5| and *p* values≤10^−4^.

b Annotation according to the NCBI website (http://www.ncbi.nlm.nih.gov).

## Discussion

Zn^2+^, Mn^2+^ and Cu^2+^ play crucial roles in bacterial cells and in the host they may invade. Moreover, both host and bacteria can modulate their responses according to concentrations of the three metal ions [46]. The purpose of this study was to obtain more knowledge on the genes involved in *E. faecalis* response to high metal ions concentrations. The analysis of the genome-wide transcriptional response of *E. faecalis* V583 to zinc, manganese and copper stresses revealed induction of genes involved in different functional categories. Particularly relevant functions were transport and binding, metabolism and cellular processes, cell envelope and signal transduction.

### Transport and binding

Metal homeostasis in bacteria is achieved by metal export and uptake systems. Some of these transporter systems were highly up- or down-regulated in our experiments. The *cop* operon (*ef0297-99*) was the highest induced operon when *E. faecalis* was grown in the presence of copper. This was expected as the encoded transporter is the main factor in copper homeostasis in *Enterococcus* [30], [33]. Notwithstanding this, it has been suggested that additional putative genes and transcriptional regulators might be able to mediate the response of *E. faecalis* to copper [33]. In fact, we observed the up-regulation of other transport genes in the presence of this metal, namely *ef1673-76*, encoding two putative ABC transporters, a gene for a hypothetical protein (*ef1674*) and a transcriptional regulator gene (*ef1676*). Some of these genes were also affected by SDS and urine [21], [23]. The function of the *ef1673-76* operon has not been elucidated, but its function as encoding a putative metal transport system for Cu^2+^ would explain its significant up-regulation upon copper stress: it was the second highest up-regulated operon, after *cop*.

The most repressed operon when *E. faecalis* was grown in the presence of zinc is *ef0055-57* encoding ZnuABC orthologues. Since it is annotated as a putative zinc uptake system, its down-regulation under high zinc concentration is expected. An operon specifying a cadmium-translocating P-type ATPase and a putative SapB family protein (*ef0758*-*9*), together with another putative cadmium-translocating P-type ATPase encoding gene (*ef1400*), were highly up-regulated under zinc stress. The low concentration of metals in urine might explain the repression of *ef1400* in urine and its up-regulation with zinc [23].

Two operons, encoding transport proteins, were affected by the three metal ions, being up-regulated with zinc and down-regulated with manganese and copper. These operons, *efaCBA* and *ef0575-78*, encode homologous proteins and were also affected by growth in the presence of urine or blood [22]–[23]. The EfaCBA proteins constitute a manganese scavenging system [34]. Our results, showing the repression of *ef0575-78* in the manganese experiments, and the fact that the same genes were up-regulated in the transcriptome experiments with blood or with urine [22], [23], two Mn^2+^-depleted environments, strengthen the supposition [34] that *ef0575-78* encode another manganese scavenging system.

Many transport systems which were differentially regulated in our experiments with metals, have not yet been proven to be involved in metal uptake or efflux. That is the case of *ef0082* gene, encoding a major facilitator ABC transporter, *ef0583-ef0584* and *fatB-ceuDCB* operons. These transport systems were also affected in urine and blood experiments [22]–[23].

### Metabolism and cellular processes

The *glnR* and *glnA* genes, which are involved in glutamine regulation and synthesis, were induced at a high zinc concentration, in this work, and repressed in blood and urine experiments [22], [23]. The fact that both genes are affected under different conditions, such as during growth in the presence of blood or urine or excess zinc, highlights the importance in *E. faecalis* of nitrogen metabolism and in particular of glutamine and glutamate levels in response to environmental changes.

The arginine deiminase (ADI) operon encodes three enzymatic steps of arginine breakdown, generating ATP. The *arcA* (*ef0104*) gene was down-regulated in zinc and copper stresses and induced in the presence of manganese. The *arcC1* (*ef0106*) gene was repressed in the presence of copper; *ef*0107 and *ef*0108 were overexpressed with manganese and repressed with copper stress. Arginine can lead to the formation of nitric oxide (NO) through the action of NO synthase. It has been described that *Giardia lamblia* ADI competes with human nitric oxide synthase by scavenging arginine from the intestinal environment in the host [47]. Even more, nitric oxide synthesis in the intestinal epithelium, which represents a host defense mechanism against pathogen infection, can be blocked by ADI [47], [48]. Therefore, ADI has a relevant role in host colonization and infection. The presence and relative concentration of metals may influence the levels of ADI operon expression, as shown from our results. One might speculate that this could ultimately influence colonization and infection of the host by *E. faecalis*.

Genes involved in the general stress response of *E. faecalis* include class-I heat shock genes *dnaK* and *groEL*, class-III heat shock genes *clpPBCEX*, the regulator *ctsR* and the general stress gene *gls24*. In our metal stress experiments, only the latter gene was slightly induced in the presence of zinc stress. Other genes encoding stress related proteins (EF0781, EF1058, EF1076, EF1084) were differentially transcribed under at least one of the metal stresses studied and *ef1058* and *ef1084* were also induced in blood [22]. Oxidative stress related genes, namely *katA* and *ef2739*, which encodes an alkyl hydroperoxide reductase, were down-regulated by copper and manganese, respectively, suggesting that high concentrations of either one of those metal ions reduces the oxidative burden related with H_2_O_2_. Of the known virulence factor-encoding genes such as *gelE*, *sprE*, the *fsr* cluster, *cylB, epa, cps, srtA, atlA and efaA*, only the last gene was differentially expressed in the presence of the studied metal cations, as discussed above.

### Cell envelope

The bacterial cell wall prevents metal ions from passively entering the cell. Yet this protective barrier must be overcome in order for the cell to meet its metal requirements [49]. Interestingly, some genes involved in the cell envelope were affected in our experiments with copper or zinc, namely those involved in the teichoic acid pathway (*ef2486-87*), found to be repressed in the presence of zinc, blood and urine [22], [23], and the *dltABCD* operon, found to be up-regulated by copper. The *dltABCD* operon is responsible for incorporating D-alanine residues into cell-wall associated teichoic acids and lipoteichoic acids (LTA). D-alanylation of lipoteichoic acids helps regulate many bacterial processes including cation regulation, activity of autolysins, and resistance to antimicrobial compounds [50]. LTA together with peptidoglycans define the polyelectrolyte properties of the cell wall and provide anionic sites for binding of metal cations. Thus, these structures are responsible for cation homeostasis and assimilation [51]. The overexpression of the referred genes in the presence of copper may help sequester the excess of Cu^2+^, preventing these ions to take part in redox reactions that would lead to the production of reactive oxygen species able to damage the cell. Also noteworthy, *ef3314* encodes a cell wall surface anchor family protein recently shown to be important for the pathogenicity of *E. faecalis* [52]. It was up-regulated in copper stress. Overall, the cell envelope seems to have a relevant role in the response to high concentrations of copper.

### Signal transduction

The genes *kdpDE*, (*ef0570-1*) constituting the two-component system *Ehk12-Err12* reported to be involved in regulation of potassium transport [42], and another two-component system, *Err06-Ehk06* (*ef1260-61*), with unknown function, were induced in the presence of zinc. In *E. faecalis* V583, the *ehk*12-*err*12 locus is located in the PAI, a transferable genetic element containing genes with known or potential roles in virulence, or adaptation and survival in different environments [53]. This is the first report on the influence of zinc on the expression of *err06-ehk06* and *ehk12-err12*.

### Summary

**Figure 4:**
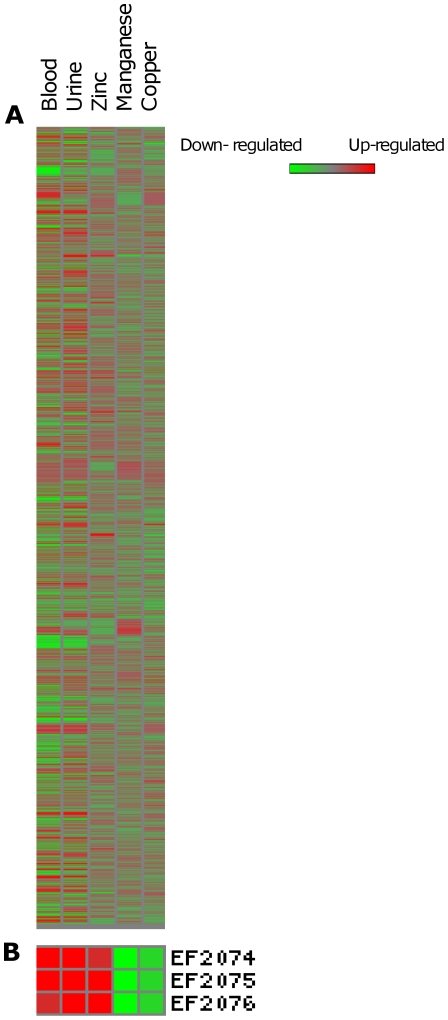
Heat map of differentially expressed genes grown in blood, in urine and with metal stresses. a) Heat map visualizing the regulated genes in the trancriptome experiments with blood [22], urine [23] and zinc, manganese and copper stresses (this work). b) Detail of the heat map showing the transcriptome results for the *efaCBA* operon. Genes found to be significantly regulated are indicated by either red (up-regulated) or green (down-regulated).

In summary, mostly transporters are involved in the response of *E. faecalis* to high concentrations of the metals ions Cu^+2^, Zn^+2^ or Mn^+2^. Also, rearrangements in the expression of genes involved in signal transduction, amino acid metabolism and related to the cell envelope may play important roles. The added metals seem to mainly induce metal-specific transcriptional responses, as there were not many differentially expressed genes in common in the three DNA microarray experiments. Most genes affected under the conditions we applied seem to be part of an *E. faecalis* response to environmental stresses and do not constitute a general stress response. This is supported by the negligible number of genes involved in DNA metabolism and encoding general stress proteins. An overall comparison of our transcriptome results with the blood and urine transcriptomes [22], [23] is represented by the heat map in Figure 4a. This representation shows the parallels between *E. faecalis* gene expression in conditions that mimic host environments such as blood and urine and in conditions with high metal concentrations. *E. faecalis* V583 transcriptome responses to the metals studied showed many differentially expressed genes in common with the referred host environments, as has been discussed throughout this paper. A clear example discussed in this paper is the low concentration of manganese ions in blood and urine, which induced the expression of Mn^2+^ transport systems and adhesion lipoproteins such as the virulence factor EfaA (Figure 4b). As shown here the same Mn^2+^ transporter systems were repressed when *E. faecalis* was confronted with a high Mn^+2^ concentration, adding support to the notion that these transporter systems are important in *E. faecalis* adaptation to the environment and for colonization and virulence. It is plausible that bacteria, in general, sense their environment in terms of its components. As mentioned before, metal ions are essential elements and bacteria need to monitor and control their levels and be able to quickly adapt to changes in these concentrations by readjusting their genome expression profile. In this sense, metal ion concentrations can be extremely important in certain environments as they can trigger or tone down the expression of genes necessary for *Enterococcus* colonization and virulence.

The broadening of our knowledge on the mechanisms that *E. faecalis* employs to maintain metal homeostasis will certainly help understand how this important human pathogen is able to adapt to diverse environments, colonize and become virulent.

## Supporting Information

Figure S1 **Expression of genes** ***ef0758*** **and** ***mntH2*** **in the presence of metals by sqRT-PCR.** The effect of metal addition on the transcription of the *E. faecalis* V583 genes *ef0758* (**A**) and *mntH2* (**B**), by semi-quantitative Reverse Transcriptase-PCR. Cells were grown in GM17 in the absence (-) or presence of added ZnCl_2_ 4 mM (Zn), MnCl_2_ 0.4 mM (Mn) or CuSO_4_ 0.05 mM (Cu). Triangles represent the decrease in cDNA concentration used in the PCR reactions (dilutions 10^0^, 10^−1^ and 10^−2^). M indicates 1 kb plus DNA ladder (Gibco, Invitrogen).
(TIF)

Figure S2 **Representation of the effect of metal addition on an** ***ef0575*** **promoter::** ***lacZ*** **fusion by β-galactosidase assays.** Representation of β-galactosidase assays showing the expression of a plasmid-encoded P*ef0575*-*lacZ* fusion in *E. faecalis* VE14089 grown in the presence of metal ions. The strain was grown in GM17 with or without one of the following added metals: ZnCl_2_, 4 mM (Zn); MnCl_2_, 0.4 mM (Mn) or CuSO_4_, 0.05 mM (Cu).
(TIF)

Table S1 **Gene expression data of *E. faecalis* V583 grown to mid-exponential phase in the presence of 4 mM ZnSO_4_, 0.4 mM MnSO_4_ or 0.05 mM CuSO_4_, relative to that during growth in GM17.** Significantly differentially expressed genes, in bold, have a *p* value≤0.0001 and a ratio≥|1.5|. “NA” denotes non-expressed or excluded genes.
(XLS)
