# Supplementary figures and images for: Impact of Manganese, Copper and Zinc Ions on the Transcriptome of the Nosocomial Pathogen Enterococcus faecalis V583
Source: PLoS One. 2011 Oct 28;6(10):e26519. doi: 10.1371/journal.pone.0026519 (PMC3203883; doi:10.1371/journal.pone.0026519)

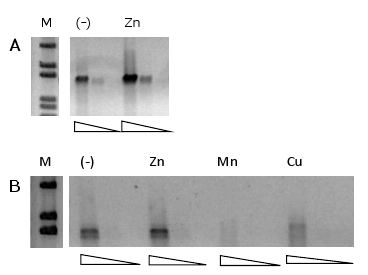

Supplement: Figure S1 — Expression of genes ef0758 and mntH2 in the presence of metals by sqRT-PCR. The effect of metal addition on the transcription of the E. faecalis V583 genes ef0758 (A) and mntH2 (B), by semi-quantitative Reverse Transcriptase-PCR. Cells were grown in GM17 in the absence (-) or presence of added ZnCl2 4 mM (Zn), MnCl2 0.4 mM (Mn) or CuSO4 0.05 mM (Cu). Triangles represent the decrease in cDNA concentration used in the PCR reactions (dilutions 100, 10−1 and 10−2). M indicates 1 kb plus DNA ladder (Gibco, Invitrogen). (TIF) [file pone.0026519.s001.tif]

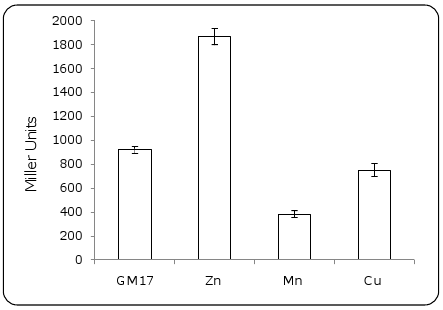

Supplement: Figure S2 — Representation of the effect of metal addition on an ef0575 promoter:: lacZ fusion by β-galactosidase assays. Representation of β-galactosidase assays showing the expression of a plasmid-encoded Pef0575-lacZ fusion in E. faecalis VE14089 grown in the presence of metal ions. The strain was grown in GM17 with or without one of the following added metals: ZnCl2, 4 mM (Zn); MnCl2, 0.4 mM (Mn) or CuSO4, 0.05 mM (Cu). (TIF) [file pone.0026519.s002.tif]
